# Expression of SV2 isoforms during rodent brain development

**DOI:** 10.1186/1471-2202-14-87

**Published:** 2013-08-09

**Authors:** Julie Crèvecœur, Patrik Foerch, Melissa Doupagne, Caroline Thielen, Catherine Vandenplas, Gustave Moonen, Manuel Deprez, Bernard Rogister

**Affiliations:** 1 Laboratory of Developmental Neurobiology, GIGA-Neurosciences, University of Liege, Sart Tilman Liege B-4000, Belgium; 2 Laboratory of Neuropathology, GIGA-Neurosciences, University of Liege, Sart Tilman, Liege B-4000, Belgium; 3 Departement of Neurology, CHU, University of Liege, Sart Tilman, Liege B-4000, Belgium; 4 Laboratory of Developmental Neurobiology, GIGA-Development, Stem Cells and Regenerative Medicine, University of Liege, Sart Tilman, Liege B-4000, Belgium; 5 UCB Pharma S.A., CNS Research, Braine-l’Alleud B-1420, Belgium

**Keywords:** SV2, Mouse brain, Development, Epileptic seizures

## Abstract

**Background:**

SV2A, SV2B and SV2C are synaptic vesicle proteins that are structurally related to members of the major facilitator superfamily (MFS). The function and transported substrate of the SV2 proteins is not clearly defined although they are linked to neurotransmitters release in a presynaptic calcium concentration-dependent manner. SV2A and SV2B exhibit broad expression in the central nervous system while SV2C appears to be more restricted in defined areas such as striatum. SV2A knockout mice start to display generalized seizures at a late developmental stage, around post-natal day 7 (P7), and die around P15. More recently, SV2A was demonstrated to be the molecular target of levetiracetam, an approved anti-epileptic drug (AED). The purpose of this work was to precisely analyze and quantify the SV2A, SV2B and SV2C expression during brain development to understand the contribution of these proteins in brain development and their impact on epileptic seizures.

**Results:**

First, we systematically analyzed by immunohistofluorescence, the SV2A, SV2B and SV2C expression during mouse brain development, from embryonic day 12 (E12) to P30. This semi-quantitative approach suggests a modulation of SV2A and SV2B expression in hippocampus around P7. This is the reason why we used various quantitative approaches (laser microdissection of whole hippocampus followed by qRT-PCR and western blot analysis) indicating that SV2A and SV2B expression increased between P5 and P7 and remained stable between P7 and P10. Moreover, the increase of SV2A expression in the hippocampus at P7 was mainly observed in the CA1 region while SV2B expression in this region remains stable.

**Conclusions:**

The observed alterations of SV2A expression in hippocampus are consistent with the appearance of seizures in SV2A−/− animals at early postnatal age and the hypothesis that SV2A absence favors epileptic seizures around P7.

## Background

SV2 proteins are integral transmembrane proteins expressed on synaptic dense core vesicles [1,2] but are also found on small clear vesicles containing neurotransmitters [3]. Cloning of the individual family members resulted in the identification of three different isoforms, SV2A [1], SV2B [4] and SV2C [5]. The overall homology between the three rat isoforms is approximately 60%, with SV2A and SV2C being more similar to each other than SV2B [5]. SV2A is ubiquitously expressed in the central nervous system (CNS), neuroendocrine cells [6,7] and at the neuromuscular junction [8]. SV2B is widely expressed in the brain [7-12] while SV2C has a more restricted distribution in the CNS [5,8,11,13,14].

All SV2 isoforms are characterized by twelve transmembrane domains and three N-glycosilation sites in the intravesicular loop [5,7,9]. SV2 proteins show similarities to members of the membrane transporter family and belong to the major facilitator superfamily (MFS), although the transported substrate has not been identified yet, and the molecular function of SV2 proteins remains elusive.

However, interactions with several synaptic proteins and hypothesis about their possible function have been reported. Botulinum Neurotoxin E was shown to interact with glycosilated forms of SV2A and SV2B [8,15]. Moreover, Mahrhold et al. reported that the carboxy-terminal region of SV2C intravesicular domain mediates the Botulinum Neurotoxin A entry leading to its toxic effects [16].

Several groups suggested a role of SV2 proteins in the regulation of presynaptic calcium concentration [17-19]. In contrast, other authors have reported that SV2 protein activities are not related to a change in presynaptic calcium [20,21]. However, there is evidence linking SV2 and calcium given the demonstrated interaction of SV2 with synaptotagmin considered to be the primary calcium sensor triggering calcium-dependent exocytosis [22-24]. More recently, Wan et al. used mouse rod bipolar cell preparations for direct biophysical studies measuring exocytosis and presynaptic calcium concentration in the mammalian central nervous system. In these neurons, SV2B is the main SV2 isoform expressed [25,26]. As SV2B knockout (KO) mice are viable [17], Wan et al. were able to show in rod cells from SV2B KO animals, an elevation of Ca^2+^ in nerve terminal both in resting and evoked presynaptic signals. This increase of Ca^2+^ concentration results in changes in synaptic vesicle dynamics, synaptic plasticity, and synaptic strength.

SV2A, the major isoform in the CNS has been linked with seizures and an epileptic phenotype. SV2A KO animals are characterized by the onset of epileptic seizures in early postnatal stage (postnatal day 7 or P7) leading to death around P15 [17,27]. Moreover, it appears that SV2A expression decreases during epileptogenesis and chronically epileptic animals [28-30] as well as in patients with temporal lobe epilepsy [31,32]. The therapeutic interest of SV2 proteins has been demonstrated with the antiepileptic drug levetiracetam. Consecutively SV2A was identified as the molecular target of the previously described brain-binding site of levetiracetam [27,29]. Levetiracetam has a unique activity profile in animal models of seizure and epilepsy, favourable side-effect profile and straightforward pharmacokinetics [33]. Furthermore, brivaracetam, a novel antiepileptic drug (AED) with higher SV2A affinity than levetiracetam [34], shows higher potency in several preclinical models of epilepsy [35] and is currently in clinical development [36].

In this study, we therefore systematically analyzed the expression of all SV2 isoforms during mouse brain development, to contribute to the understanding of their role during development and the onset of epileptic seizures.

## Results

### Immunohistofluorescence (non quantitative)

A systematic analysis of immunoreactivity for SV2A, SV2B and SV2C in several brain regions at various ages was performed. The results obtained for the selected telencephalic regions are summarized in Table 1. It was previously demonstrated that SV2A KO mice started to experience epileptic seizure at P7. Thus, the expression of all SV2 isoforms was analyzed at different key developmental steps, focusing on the developmental period around P7. In grey matter of cortices, SV2A labelling was detectable from embryonic day 14 and reached maximum expression at post-natal day 9 (P9). In olfactory bulb, the signal for SV2A reached a peak at embryonic day 16 (E16) and remained stable in both fascicular and glomerular regions up to post-natal day 30 (P30). In hippocampus, the labelling was detected earlier, at embryonic day 14 (E14) but remained localized in the hilus of *dentatus gyrus* (DG). The signal in DG seemed to decrease transiently around P7 and then increases in this region to reach maximum expression at P10. In the CA1 region, low level of SV2A expression was detectable from P7 to reach maximum levels in older animals (P30). At P7, the signal remained stable in the olfactory bulbs, indicating that the decrease observed around P7 in hippocampus was not due to technical issues related to immunolabelling but truly reflects reduced levels of SV2A in hilus of DG at P7. However, if we consider the growth of these two structures (hippocampus and olfactory bulb), one can also observe a higher expansion of the hippocampus than the olfactory bulb around P7. In sub-cortical nuclei and in pallidal regions, SV2A signal appeared between embryonic day 14 and 16 (E14 and E16) and rapidly reached high expression intensities. In these regions the signal also seemed to decrease around P7 but this decrease was less pronounced than what is observed in hippocampus. The results for the diencephalic, mesencephalic, pontic, bulbar and cerebellar regions are summarized in the Additional files 1 and 2.

**Table 1:** Expression levels of SV2A in various telencephalic regions at various ages (embryonic day 12, 14, 16, 18 and post-natal day 0, 1, 6, 7, 8, 9, 10, 15 and 30)

***TELENCEPHALIC REGIONS***	**E12**	**E14**	**E16**	**E18**	**P0**	**P1**	**P6**	**P7**	**P8**	**P9**	**P10**	**P15**	**P>/= 30**
**Cerebral Cortex**													
Grey matter	-	++	++	++	++	++	++	++	++	+++	+++	+++	+++
White Matter	-	-	-	-	-	-	-	-	-	-	-	-	-
**Olfactory Bulb**													
Fascicular region	-	-	+	++	++	+++	+++	+++	+++	+++	+++	+++	+++
Glomerular region	-	-	+++	+++	+++	+++	+++	+++	+++	+++	+++	+++	+++
**Hippocampus**													
CA1	-	+	+	+	-	-	-	+	+	+	+	+	+++
Dentatus Gyrus	-	-	-	-	-	+	-	-	-	-	-	-	+++
Hilus of dentatus gyrus (CA4)	-	+++	+++	++	+++	+++	+++	+	++	++	+++	++	+++
**Sub-cortical nuclei**													
Dorsal Striatum (Caudatus N. and putamen)	-	+++	++	++	+++	+++	+++	++	++	+++	+++	++	+++
Ventral Striatum	-	-	-	++	+++	++	+++	++	+++	+++	+++	++	+++
Nucleus Accumbens	-	-	+++	++	+++	++	++	+	++	++	+++	+++	+++
Septal lateral nucleus	-	-	+++	++	+++	+++	+++	+	++	+	+++	++	+++
Septo-fimbrial nucleus	-	-	+++	++	++	+++	+++	++	+	+	+++	++	+++
Amygdala	-	++	+++	+++	+++	+++	+++	+	+++	+++	+++	++	+++
**Pallidum**													
Dorsal pallidum	-	-	-	-	+++	+++	+++	++	+++	+++	+++	++	+++
Ventral pallidum	-	-	-	-	+++	++	+++	++	++	+	+++	++	+++
Magno-cellular nucleus	-	-	+++	+++	++	+++	+++	++	++	+	+++	++	++
Posterior Pallidum (stria terminalis nucleus)	-	++	+++	++	+++	+++	+++	++	+++	++	+++	++	+++
***DIENCEPHALIC REGIONS***													
**Thalamus**													
Polymodal sensory thalamus	-	-	-	-	+++	+++	++	+	+++	+	++	++	++
Anterior thalamus	-	+++	++	++	++	++	++	+	+++	++	++	+++	++
Lateral thalamus	-	-	-	++	++	+++	+++	++	++	+	++	++	++
Ventral thalamus	-	-	-	1++	+++	+++	+++	++	+++	++	+	++	+++
Intralaminar thalamic nuclei	-	-	-	-	+++	+++	+++	+	++	++	+	++	+++
Habenula	-	-	+++	++	+++	+++	+++	++	+++	++	+++	+++	+++
Medio-dorsal thalamus	-	-	-	++	++	+++	++	+	+++	+	++	++	+++
Geniculated nuclei	-	+++	+++	+	+++	+++	+++	++	+++	+	+++	+++	+++
**Hypothalamus**													
Periventricular region	-	-	++	++	+++	+++	+++	+++	+++	+	+++	+++	+++
Arcuetus nucleus	-	-	+++	+++	+++	+++	+++	+	+++	++	+++	+++	+++
Paraventricular nucleus	-	-	+++	+++	+++	+++	+++	++	+++	+	++	+++	++
Paraventricular region	-	-	++	+++	+++	+++	+++	+	+++	+	++	++	+++
Pre-optic nucleus	-	+++	++	++	+++	+++	+++	++	+++	+	+++	+++	+++
Supra-chiasmatic nucleus	-	-	++	++	+++	+++	+++	++	+++	++	+++	+++	+++
Medial Hypothalamus	-	-	-	-	+	+++	+++	++	+++	+	++	+++	++
Mamillary bodies	-	-	++	++	+++	+++	+++	+	+++	++	+++	++	+++
***MESENCEPHALIC REGIONS***													
**Sensory Mesencephale**													
Inferior colliculus	-	-	-	+++	+++	+++	+++	+++	+++	++	++	+++	+++
Superior colliculus	-	-	++	++	+++	+++	+++	+++	+++	++	++	+++	+++
**Motor Mesencephale**													
Substantia nigra	-	+++	+++	+++	+++	+++	+++	++	+++	++	+++	+	+++
Ventral tegmental area	-	-	++	+++	+++	+++	+++	++	++	+++	+++	++	++
Peri-aqueducal grey matter	-	-	+++	+++	+++	++	+++	+++	+++	+++	+++	++	+++
Pretectal region	-	-	+++	+++	+++	+++	+++	++	+++	+++	+++	++	++
Red nucleus	-	-	+++	++	+++	+++	+++	++	+++	+++	++	++	+++
Cuneiform nucleus	-	-	-	-	+++	++	+++	++	+++	++	+++	++	++
Common oculomotor nucleus (III)	-	+++	+++	+++	+++	+++	++	+++	+++	+	+++	+++	+++
Edinger Westphal nucleus	-	-	-	-	+++	+++	+++	++	+++	+++	++	++	+++
**Behavioural Mesencephale**													
Pedoculo-pontic nucleus	-	-	-	-	+++	+++	+++	+++	+++	+++	+++	+++	+++
Raphe nuclei	-	-	++	+++	+++	+++	+++	+++	++	+++	++	++	+++
***PONTIC REGION***													
**Sensory pons**													
Lateral lemniscus nucleus	-	-	+++	+++	+++	+++	+++	++	+++	++	+++	++	+++
Superior olivary tractus nucleus	-	-	+++	+++	+++	+++	+++	+++	+++	++	++	+	+
**Motor Pons**													
Motor nucleus of V	-	-	++	++	+++	+++	+++	+++	+++	+++	+++	+	+++
Facial nucleus (VII)	-	+++	+++	+++	+++	+++	+++	++	+++	+++	++	+	+++
Dorsal tegmental nucleus	-	-	-	-	+++	++	+++	++	++	++	+++	+	+++
**Behavioural Pons**													
Raphe nuclei	-	-	++	+++	+++	+++	+++	+++	++	+++	++	++	+++
***BULBAR REGION***													
**Sensory bulb**													
Cochlear nucleus	-	-	+++	+++	+++	+++	+++	++	+++	+++	+++	+++	+++
Cuneus nucleus	-	-	+++	+++	+++	+++	+++	+++	+++	+++	+++	++	++
Solitary tractus nucleus	-	-	+++	+++	+++	+++	++	++	+++	++	++	++	+++
**Motor bulb**													
Ambiguus nucleus	-	+++	+++	+++	+++	+++	+++	++	+++	+++	+++	++	+++
Dorsal nucleus of vagal nerve	-	-	+++	+++	+++	+++	+++	++	+++	+++	+++	++	++
Inferior olivary complex	-	-	++	+++	+++	+++	+++	++	+++	++	++	++	++
Vestibular nucleus	-	+++	+++	+++	+++	+++	+++	++	+++	++	+++	++	+++
Hypoglosse nucleus	-	+++	+++	+++	+++	+++	+++	+++	+++	++	++	++	+++
***CEREBELLUM***													
**Cerebellar cortex**													
Molecular layer	-	-	-	-	+++	+++	+++	+++	+++	+++	+++	+++	+++
Purkinje cells layer	-	-	-	+++	+++	+++	+++	+++	+++	+++	+++	+++	+++
Internal granular layer	-	-	-	-	-	-	-	-	-	-	-	-	-
**Cerebellar nuclei**													
Dentatus nucleus	-	-	-	++	+++	+++	+++	+++	+++	+++	+++	++	++
Fastigial nucleusl	-	-	-	+++	+++	+++	+++	+++	+++	+++	+++	+++	+++

The level of immunohistofluorescence has been evaluated based of the signal intensity in four classes : - for no labelling, +, ++ and +++ for respectively low, medium and intense fluorescence and brightness.

### Quantitative confocal immunofluorescence

**Figure 1:**
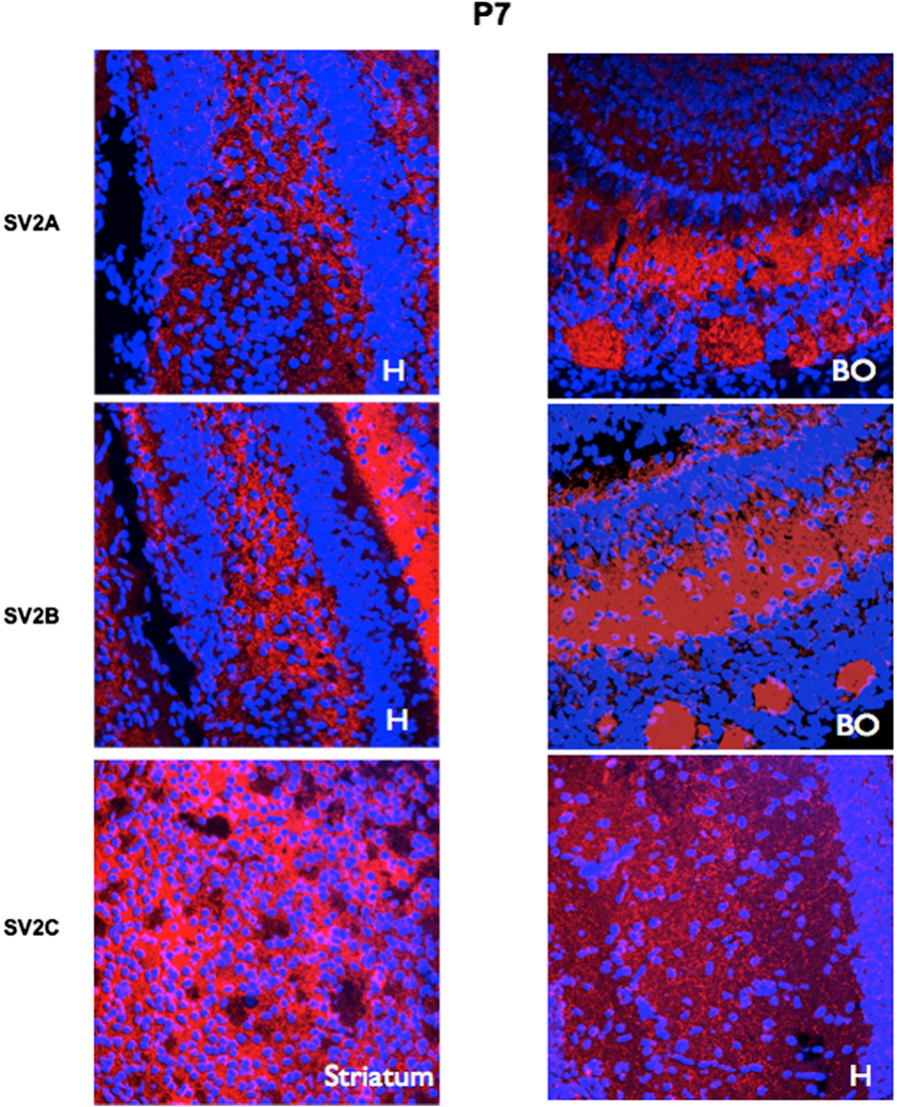
**Immunofluorescence of SV2A, SV2B and SV2C in mouse brain at post-natal day 7 (P7).** Fluorescent images of SV2A, SV2B and SV2C labeling in the hippocampus (H), olfactory bulb (BO) and striatum at P7. Five animals per age were observed in sagittal sections, 4 or 5 animals per age in coronal sections. Nuclei were counterstained with DAPI (blue). Original magnification 40X.

**Figure 2:**
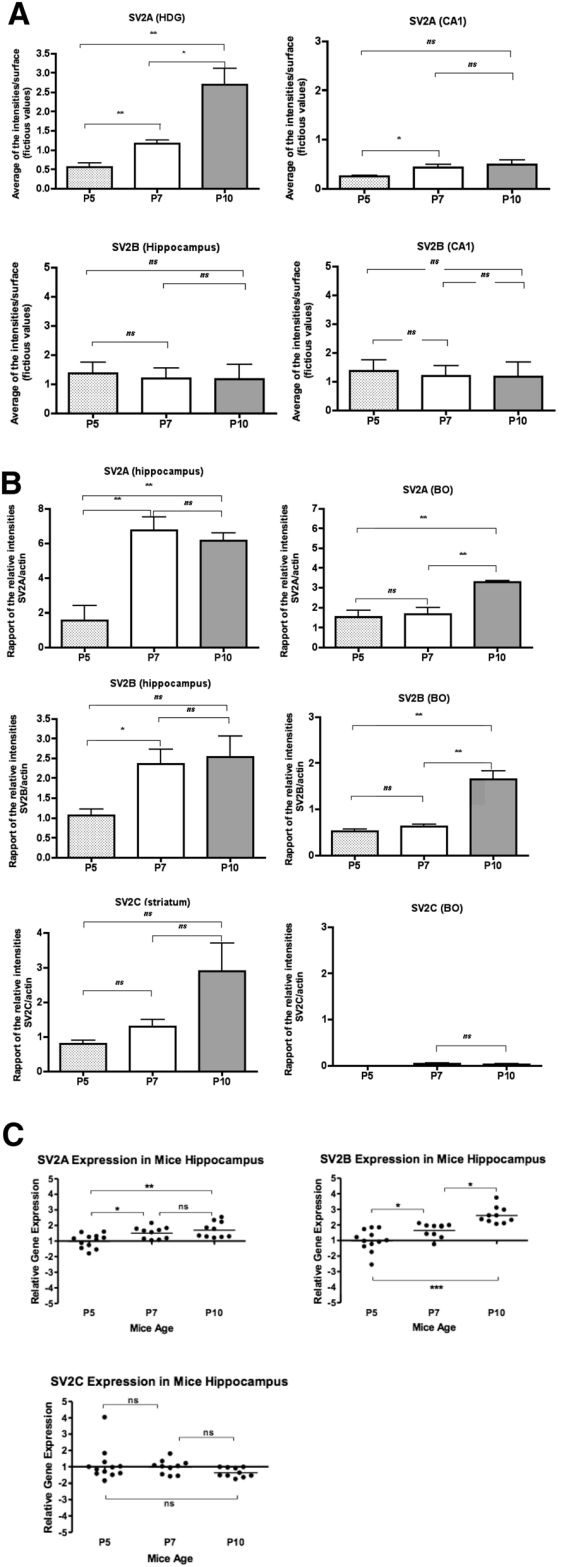
**Quantification of SV2 isoforms. A**: Quantification of SV2A and SV2B in mouse brain by confocal microscopy at P5, P7 and P10. Quantification by confocal microscopy were perfomed by using Olympus software F10 ASW, allowing to measure the intensity of SV2A and SV2B compared to selected surface. Different regions were selected for these measures: hippocampus, hilus of dentatus gyrus (HDG) and CA1of mice at P5, P7 and P10. Three animals per age were used this quantification. Data are presented as mean +/− SD and were analyzed by ANOVA following a Student’s *t* test. * p < 0.05; **p < 0.01; ns: not significant. **B**: Quantification of SV2A, SV2B and SV2C in mouse brain by western blot. Fluorescent western blots analysis of SV2A, SV2B and SV2C proteins were carried out on hippocampus, striatum and olfactory bulb (BO) of mice at P5, P7 and P10. Three animals per age were used. Quantification of these western blots was performed using software Image Master 1D Elite. Data are presented as mean +/− SD and were analyzed by ANOVA following a Student’s *t* test. * p < 0.05; **p < 0.01; ns: not significant. **C**: Quantification of SV2A, SV2B and SV2C mRNA in mouse hippocampus. mRNA expression levels were measured by RT-qPCR and normalized to the housekeeping gene β-actin. Data were analyzed by a one-way ANOVA followed by a Bonferroni’s Multiple Comparison Test. Three animals per age were used this quantification. This experiment was repeated once with three new animals per age. * p < 0.05; **p < 0.01; ***p < 0.001; ns: not significant.

An apparent reduction of SV2A immunolabeling was thus observed in hippocampus at P7, which is exactly the period at which SV2A KO mice display epileptic seizures. In order to understand whether reduced SV2A expression in hippocampus – correlating with seizure appearance – was secondary to hippocampal growth which is pronounced at that age, we decided to quantify more precisely the expression of SV2A at post-natal day 5, 7 and 10 (Figure 1). In addition, expression of SV2B and SV2C was studied in the same regions (Figure 1). First, we used the fluorescence index quantified using confocal microscopy for CA1 region and hilus of DG. As signals for SV2A in olfactory bulbs remained stable from P5 to P10 (Table 1), both signals in CA1 and hilus of DG were normalized using the signal in olfactory bulb to minimize the variation due to the immunolabeling technique itself (Figure 2A). SV2A immunohistofluorescence increased gradually in the hilus of DG between post-natal days 5 (P5) and 10 (P10) while in CA1 region, the signal for SV2A increased only between P5 and P7. Albeit statistically significant the level of increase in the CA1 was substantially lower than the one observed in the hilus of DG. In contrast, the SV2B signal was not altered during this period both in CA1 and in the hilus of DG. Finally, SV2C was not detected in the hippocampus at these three ages but expression was confirmed in striatum (data not shown). Therefore, the results obtained by quantified confocal microscopy for SV2A in hippocampus did not support the decrease of SV2A which was first suspected using a non-quantified approach.

### Laser microdissection and quantitative Western blot

We further used quantitative western blot to determine levels for SV2A and SV2B at P5, P7 and P10. Hippocampus and olfactory bulbs were microdissected and proteins were extracted, quantified and analyzed by western blot using actin as reference for normalization between samples (Figure 2B). Both SV2A and SV2B expression increased between P5 and P7 in the entire hippocampus and remained stable between P7 and P10. In contrast, SV2A and SV2B expression in olfactory bulbs remained constant between P5 and P7 but significantly increased between P7 and P10. SV2C was expressed at very low level in hippocampus (data not shown) and olfactory bulbs (Figure 2B). In the striatum, where this isoform is highly abundant, an increase was observed between P7 and P10. These data correlated with the results obtained using qRT-PCR on mRNA extracted from hippocampus: SV2A mRNA levels expressed in the hippocampus showed a significant increase between P5 and P7 and P10 respectively. Similarly a significant increase of SV2B mRNA level was detected in the hippocampus at P10 and P7 relative to mRNA quantified at P5. In contrast, the SV2C mRNA expression was not altered and remained stable albeit at a substantially lower level relative to SV2A and SV2B at all time points (Figure 2C). Protein levels measured by western blot and mRNA data analyzed by qPCR were consistent for majority of time points and hippocampal sub-regions except for the increase of SV2B mRNA levels in the whole hippocampus between P7 and P10 with only a limited increases for SV2B protein expression in western blot.

**Figure 3:**
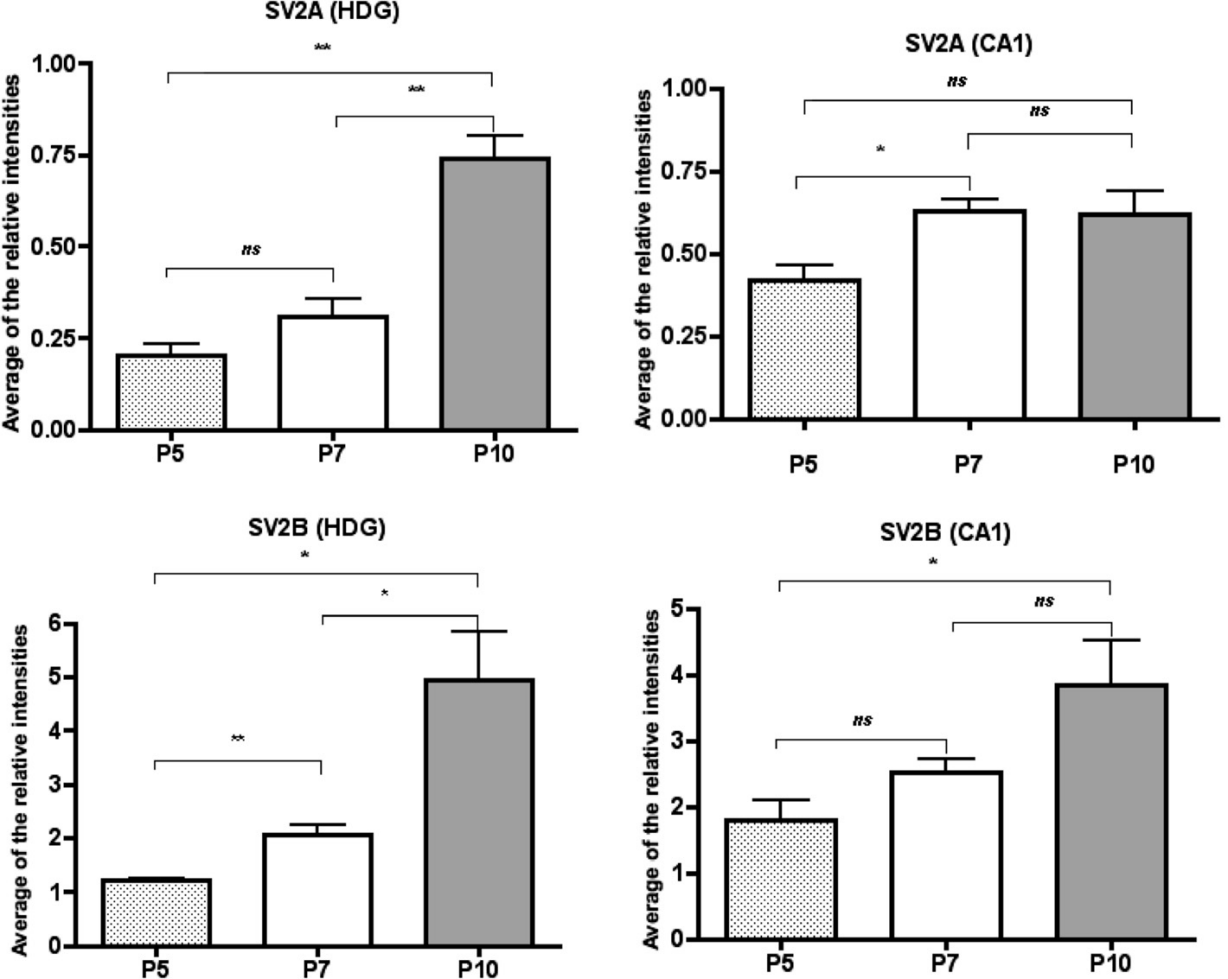
**Quantification of SV2A and SV2B by western blots in laser micro-dissection hippocampal regions.** Fluorescent western blot analysis of SV2A and SV2B proteins were carried out on the hilus of dentatus gyrus (HDG) and the CA1 region laser micro-dissected from the hippocampus of mice at P5, P7 and P10. Three animals per age were used this quantification. This experiment was repeated once with three new animals per age. The quantification was performed using software Image Master 1D Elite. Data are presented as mean +/− SD and were analyzed by a ANOVA following a Student’s *t* test. * p < 0.05; **p < 0.01; ns: not significant.

To quantitatively assess expression of SV2 isoforms at P5, P7 and P10 in CA1 and hilus of DG of the hippocampus, laser-microdissection was performed. The proteins were extracted and western blotting signals were quantified (Figure 3). Using quantitative western blotting, we confirmed that in the hilus of DG, SV2A expression increased between P5 and P7 and between P7 and P10, albeit statistical not significant for the earlier timepoint. In the CA1 region of the hippocampus, the intensity of SV2A signal increased between P5 and P7 and remained stable at P10. For SV2B, an increase of expressed protein was detected in the hilus of DG between P5 and P7 and between P7 and P10. In the CA1 region, SV2B protein level increased between P5 and P10 but no significant difference between P5 and P7 as well as P7 and P10 was detected.

## Discussion

Our study focuses on the expression of the three SV2 protein isoforms in the mouse developing brain from embryonic day 12 (first differentiated neurons) to post-natal day 30 (P30). We analyzed several regions in the central nervous system, focusing the analysis of the SV2 protein expression in telencephalic regions given the relationships of SV2A with epilepsy. Interestingly, non quantitative immunofluorescence suggests a decrease of the SV2A signal in the hippocampus around P7 which is precisely the age at which epileptic seizures are observed in SV2A KO mice. A decrease of the SV2A signal was also observed using this semi-quantitative approach at P7 in various telencephalic regions (sub-cortical nuclei and pallidum), including the thalamus, as well the hypothalamus, the mesencephalon in the pontic and the bulbar regions suggesting potentially a technical reason for this reduction. However, this technical problem is unlikely as the decreased SV2A signal at P7 was not observed in other brain regions such as olfactory bulbs or in the cortical grey matter. Interestingly, the growth of all these telencephalic structures are characterized by a rapid expansion at P7 given the myelinogenesis which takes place at that age [37]. For this reason, we performed a precise quantification of the SV2A protein and mRNA expression levels in various hippocampal regions around P7. This precise quantification did not confirm the apparent reduction of SV2A at P7 observed by immunohistology. Similarly, a systematic analysis of SV2B expression in various brain regions by the same methodological approach gave similar results: an apparent decreased expression of SV2B in hippocampus around P7 (data not shown) which was not confirmed by the quantitative SV2B expression measurements. Thus, we believe that this decrease in SV2A and in SV2B expression which is apparent for hippocampus, is a consequence of a phenomenon which is not related to the SV2A or SV2B expression per se but rather to the enlargement and growth of tissue as myelin appears at that age in these various mouse brain regions [37], potentially affecting the semi-quantitative immunohistological read out suggesting a decrease in SV2A or SV2B expression.

A more precise quantification of SV2A, SV2B and SV2C in whole hippocampus showed an increase in the expression of SV2A and SV2B between P5 and P7, and a stabilization between P7 and P10, while SV2C showed low expression at P5, P7 and P10. Majority of the protein quantification data were confirmed by mRNA measurements using qPCR indicating elevated levels for SV2A and SV2B at later stages except for SV2B mRNA which was increased between P7 and P10. The increase of SV2A expression in the hippocampus observed by confocal microscopy measurements, between P5 and P7 seems to be restricted exclusively to the CA1 region. Indeed, we confirmed a higher SV2A expression by western blot in the CA1 after laser micro-dissection. The SV2A expression in hippocampus is thus different than the SV2B expression which progressively increases in CA1. An observation that could possibly explain that the absence of SV2A in KO mice at that age is followed by onset of epileptic seizures onset, while the absence of SV2B has no effect on seizure susceptibility [38,39]. In this context, as presynaptic calcium regulation by SV2 proteins have been hypothesized by studying SV2B KO mice, the question of the exact role of SV2A, the target of the anti epileptic drug levetiracetam, however remains despite intense research elusive.

We thus observed a sharp increase of SV2A expression in CA1 region at P7, the age of onset of epileptic seizures in SV2A KO animals [17]. SV2A is expressed as early as neurons differentiated, the question of the appearance of seizures only at a late developmental stage has to be addressed. On the other hand, the role of SV2A in seizure susceptibility could be linked to its presence in inhibitory neurons. When the seizures appear, GABA is changing from being an excitatory to an inhibitory transmitter [40]. It was previously described, that P7 is the earliest developmental stage where a long-term potentiation in CA1 region in rats can be demonstrated suggesting an increase of synaptic plasticity at that age [41]. More recently, it has been demonstrated that levetiracetam controls more efficiently epileptic seizures when SV2A is highly expressed in tissue surrounding resected glioma [42]. This observation together with our study suggests that a high level of SV2A expression is required to protect animals from epileptic seizures and for the efficacy of levetiracetam. This conclusion is consistent with the observation that SV2A (+/−) heterozygous mice are more prone to seizures in a range of various acute models as well as accelerated epileptogenesis [43,44]. Starting at P7, an age characterized by myelinogenesis in several telencephalic regions and by synaptic plasticity in CA1 region, a minimum SV2A level is required to protect animals from seizures. This potential “protective” effect of SV2A increased expression in CA1 at P7 is not compensate by SV2B either because SV2A exhibit specific function(s) different to SV2B or because SV2B expression does not increase in hippocampus at the same age. In this context, it can be speculated that levetiracetam could potentially increase the SV2A activity, whatever this activity is and consequently, thereby decreasing epileptic seizures. This putative role of levetiracetam is also sustained by the observation that brivaracetam, a compound which binds SV2A with a higher affinity than levetiracetam, exhibits more potent anticonvulsant properties in various acute epilepsy models [45].

## Conclusions

In this work, we study the pattern of SV2 isoforms expression during mouse brain development. By non-quantitative and quantitative approaches, we show an increased expression of SV2A restricted to the CA1 region at P7. These findings provide new elements to better understand the role of SV2A in the epileptic seizures.

## Methods

### Animals

In this study, we used mice (BALB/c) at different ages. Validation of SV2A, SV2B and SV2C antibodies were performed on SV2A KO, SV2B KO and SV2C KO mice. The SV2C KO mice were generated in UCB (UCB Pharma SA, Braine l’Alleud). Double SV2A/SV2B KO mice were purchased from Jackson® Laboratory (Bar Harbor, Maine USA) [17]. Animal care was in accordance with the declaration of Helsinki and followed the guidelines of the Belgium ministry of agriculture in agreement with European Community laboratory animal care and use regulation (86/609/CEE, CE of J n°L358, 18 December 1986). Experimental researchs on animals were performed with the approval of ethics committee of the University of Liège. The number of the file from ethics committee is 1122 and accepted December 21, 2010.

### Numbers of animals

Regarding the results presented in the section entitled “Immunohistofluorescence (non quantitative)”, 5 animals per age were observed in sagittal sections, 4 or 5 animals per age in coronal sections. For the section entitled “Quantitative confocal immunofluorescence” 3 animals per age were used (Figure 2A). For the section entitled “Laser micro dissection and quantitative Western blot” (Figure 2B), 3 animals per age were used. In the Figure 2C and Figure 3, 3 animals per age were used this quantification. This experiment was repeated once with three new animals per age.

### Processing of tissue and sections

Mice were anaesthetized with a Nembutal® injection (Pentobarbital 60mg, Ceva Sante Animal®, Bruxelles, Belgium) before intracardiac perfusion with NaCl 0.9% (VWR International®, Prolabo), followed by paraformaldehyde (PAF) 4% (4.3g/l NaOH, 44g/l paraformaldehyde, 18.8 g/l NaH_2_PO_4_) at 4°C. Brains were removed and postfixed in PAF 4% at 4°C over night (o/n), then cryoprotected for 48h in azide phosphate buffer saline (PBS) solution containing 30% sucrose before freezing at −80°C in a 2-methylbutane solution (Aldrich®, Germany). Forty micrometer thick coronal and sagittal sections were cut on a cryostat and stored at −20°C.

### Immunolabelling and histology

Permeabilization and blocking of unspecific binding sites were performed by incubation at room temperature (RT) during 30 min in the blocking solution (10% donkey serum and 0.3% Triton X-100 in phosphate buffer saline, PBS). Primary antibodies were diluted in a solution containing 10% donkey serum (Jackson Immunoresearch Laboratories®, West Grove, PA, USA) and 0.1% Triton X-100 in PBS (carrier solution). Commercially available antibodies directed against SV2A (1:200, Abcam®, Cambridge, UK), SV2B (1:500, SYSY®, Göttingen, Germany) and SV2C (1:500, SYSY®) were used. Brain sections or fixed cells were incubated with primary antibodies at RT for 2h or at 4°C o/n. Three 15 min washes were performed in PBS at RT. rhodamine-Red-X or RRX-conjugated secondary antibodies were used (Jackson Immunoresearch Laboratories®). All secondary antibodies were diluted 1:500 in the carrier solution. Finally, tissue sections were washed three times with PBS and coverslip added using DAPI-containing Vectashield® solution (Hard Set Mounting Medium®, Vector laboratory, Burlingame, CA, USA). The slides were stored in the dark at 4°C. Selectivity was confirmed by absence of staining on slices from SV2A KO, SV2B KO and SV2C KO mice. In addition, the specificity of the antibodies was confirmed by blocking peptides (Abcam® for SV2A peptide and SYSY® for SV2B and SV2C peptides). Incubation in absence of primary antibody resulted in a complete loss of detectable signal.

### Image acquisition data analysis

Immunostained sections were examined using the Olympus Fluoview FV1000 confocal microscope (Olympus® Europa, GmbH, Hamburg, Germany). The level of immunohistofluorescence was semi-quantitatively assessed based on the signal intensity and scored in four classes (no labelling, low, medium and intense fluorescence and brightness) by two independent blinded observers. The quantification by confocal microscopy was performed using Olympus software F10 ASW.

### Western blotting

Non-fixed mouse brain was cut using a vibratome (Leica-microsystems®, Grand-Bigard, Belgium) to obtain slices. The microdissection of the slices were used to dissect region of interest in the hippocampus. Protein extraction was performed on whole tissue using lysis buffer (Triton X-100, PBS 0.1 M, NaCl 1.5 M, EDTA 0.5 M). After incubation for 15 min on ice, the lysate was centrifuged for 10 min at 4°C at 10 000 G. Supernatant was collected and stored at −80°C for western blots analysis. Samples were diluted in loading buffer (Tris 106 mM, Tris base 141 mM, LDS 2%, Glycerol 10%, EDTA 0,51 mM, SERVA Blue G250 0.22 mM and red Phenol 0.175 mM, pH: 8.5) and boiled for 5 min. Microdissected hippocampus were directly added to the loading buffer and incubated for 30 min in an ultrasonic bath. Samples were then boiled for 10 min. Proteins were separated using a 10% polyacrylamide commercial gel (NuPage®, Invitrogen®, Merelbeke, Belgium) for 55 min at 200 volts (buffer: MOPS 50 mM, Tris Base 50 mM, SDS 0.1% and EDTA 1 mM, pH: 7.7) and transferred on a PVDF membrane (Roche®, Basel, Switzerland) for 60 min at 30 volts (buffer: Bicine 50 mM, Tris Base 50 mM and SDS 0.1%, pH 8.24). Membrane blocking was performed by incubation for 1 h in a blocking solution (0.2% I-Block in PBS-Tween, Tropix®). Then, the membranes were incubated for 2 h at RT in the presence of primary antibody directed against SV2A (1:2000, Abcam®), SV2B (1:10000, SYSY®) or SV2C (1:5000, SYSY®). After three washing steps in PBS-Tween solution (Tris 50mM, NaCl 120mM and Tween 0.2%, pH 7.6), the membranes were incubated for 1h with a secondary fluorescent antibody (Cy-5 conjugated anti-rabbit IgG, Jackson Immunoresearch Laboratories®) at RT. After three additional washes, the membranes were incubated at 37°C for 1 h in the dark in SuperSignal West Pico Chemiluminescent Substrate (Thermo Fisher Scientific®, Erembodegem, Belgium). Western blots were revealed using typhoon 9400 (Amersham Pharmacia Biotech®, GE Healthcare Lifesciences, Diegem, Belgium). The quantification of western blots was performed by Image Master 1D Elite software (Amersham Pharmacia Biotech®, GE Healthcare Lifesciences).

### RNA purification and quantification

RNA extraction was performed with the RNeasy (Qiagen®, Venlo, Netherlands). One μg of total RNA was used to synthesize cDNA with the Applied Biosystems high capacity cDNA reverse transcription kit in a total volume of 100 μl following the manufacturer’s protocol (Life Technologies Corporation®, Carlsbad, California). Taqman Real-Time Quantitative PCR reactions (qPCR) were performed with the ABI 7900HT Sequence Detection System. Undiluted, 10× and 100× diluted cDNA were analyzed in duplicate for SV2A, SV2B and SV2C expression, using inventoried (SV2A and SV2B) and made to order (SV2C) Applied Biosystems TaqMan gene expression assays. Cq values were obtained (Applied Biosystems® SDS 2.3 software) using automatic threshold and baseline. To normalize the Cq values to the amount of cDNA per well (ΔCq), mouse β-actin was used as endogenous control. Relative gene expression was calculated with the formula 2^–ΔΔCq^[46], with the mean of the ΔCq of the P5 samples as calibrator to obtain the ΔCq.

### Statistical analysis

All numerical analysis were performed using GraphPad Prism software. Statistical analysis was performed using two-way analysis of variance (ANOVA) followed by a Student’s *t* post-test. Data are presented as mean with standard deviation of mean (Mean +/− SD). p value < 0,05 was considered significant. For qPCR: One-way ANOVA followed by a Bonferroni’s Multiple Comparison Test was used.

## Abbreviations

AED: Anti-epileptic drug; ANOVA: Two-way analysis of variance; CNS: Central nervous system; DG: Dentatus gyrus; E12: Embryonic day 12; KO: Knock-out; MFS: Major facilitator superfamily; P7: Post-natal 7; PBS: Phosphate buffer saline; RT: Room temperature; SV2: Synaptic vesicle protein 2.

## Competing interests

CV and PF are employees of UCB Pharma SA. The authors declare that they have no competing interests.

## Authors’ contributions

JC, PF, MeD, CV and BR carried out the studies and participated in the data analysis. JC, PF, BR drafted the manuscript. CT, GM and MaD helped in the last version of the manuscript. GM contributed to the conception and design of the project. BR conceived the study, participated in its design and coordination. All authors read and approved the final manuscript.

## Supplementary Material

Additional file 1 **Validation of antibodies anti- SV2A, SV2B, SV2C on WT,SV2A KO (SV2A−/−) and SV2B KO (SV2B−/−) mice at P7. Representative fluorescence images of SV2A, SV2B and SV2C labelling in the hippocampus (H) of WT, SV2A KO (SV2A−/−) and SV2B KO (SV2B−/−) mice at P7. Nuclei were counterstained with DAPI (blue).** Original magnification 40X. Click here for file

Additional file 2 **Validation of antibodies anti- SV2A, SV2B, SV2C on SV2A KO (SV2A−/−) mice and using blocking peptide.** Fluorescent images of SV2A labelling in the hippocampus (H) of WT, SV2A KO (heterozygous) or SV2A KO (homozygous) mice. For SV2B and SV2C, blocking peptides were used at different concentration (1 ng/mL; 10 ng/mL; 100 ng/mL; 1000 ng/mL). Nuclei were counterstained with DAPI (blue). Substantia Nigra (SN). Original magnification 40X. Click here for file
