# Supplementary figures and images for: Expression of SV2 isoforms during rodent brain development
Source: BMC Neurosci. 2013 Aug 9;14:87. doi: 10.1186/1471-2202-14-87 (PMC3765414; doi:10.1186/1471-2202-14-87)

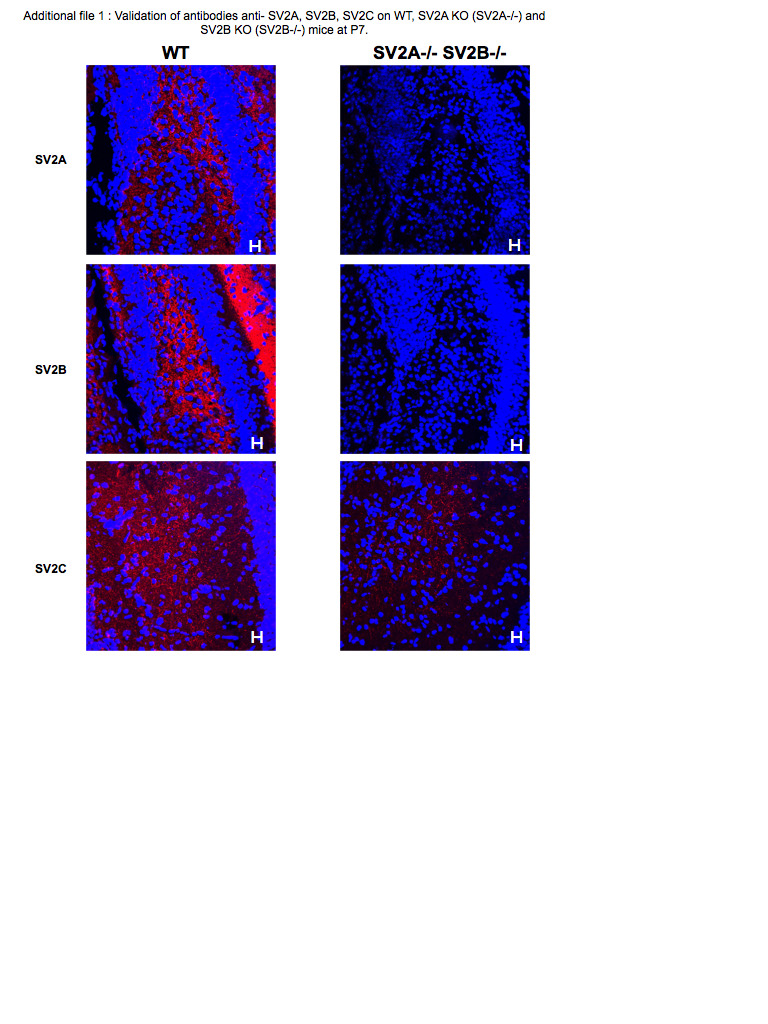

Supplement: Additional file 1 — Validation of antibodies anti- SV2A, SV2B, SV2C on WT,SV2A KO (SV2A−/−) and SV2B KO (SV2B−/−) mice at P7. Representative fluorescence images of SV2A, SV2B and SV2C labelling in the hippocampus (H) of WT, SV2A KO (SV2A−/−) and SV2B KO (SV2B−/−) mice at P7. Nuclei were counterstained with DAPI (blue). Original magnification 40X. [file 1471-2202-14-87-S1.tiff]

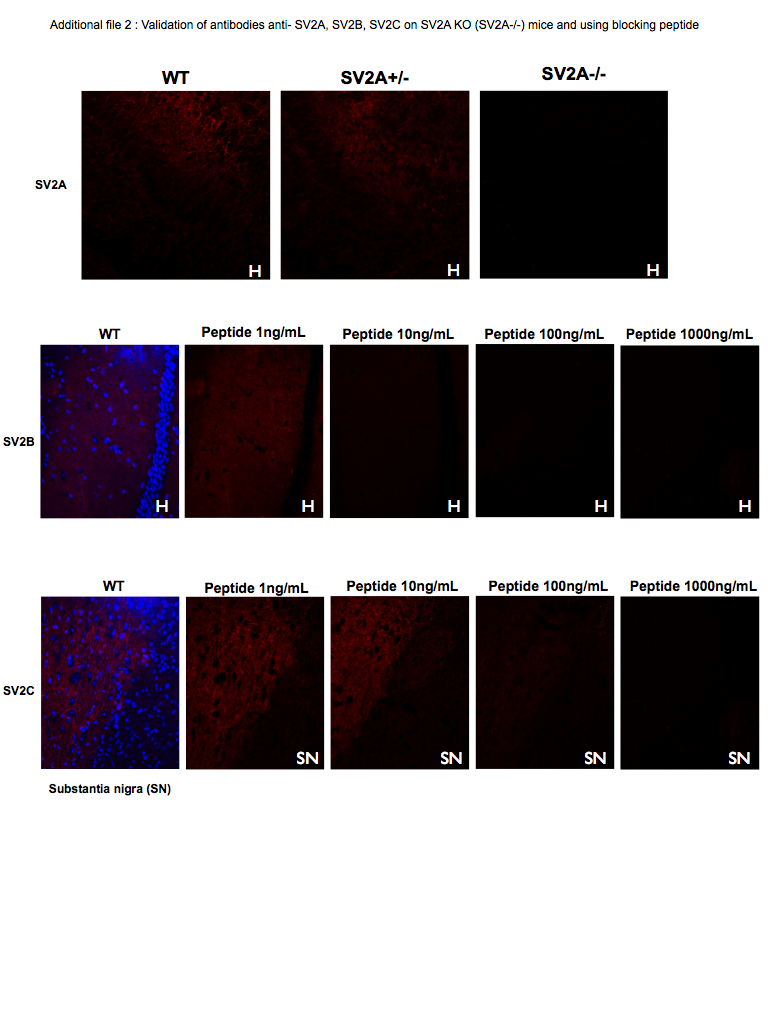

Supplement: Additional file 2 — Validation of antibodies anti- SV2A, SV2B, SV2C on SV2A KO (SV2A−/−) mice and using blocking peptide. Fluorescent images of SV2A labelling in the hippocampus (H) of WT, SV2A KO (heterozygous) or SV2A KO (homozygous) mice. For SV2B and SV2C, blocking peptides were used at different concentration (1 ng/mL; 10 ng/mL; 100 ng/mL; 1000 ng/mL). Nuclei were counterstained with DAPI (blue). Substantia Nigra (SN). Original magnification 40X. [file 1471-2202-14-87-S2.tiff]
